# *Blastocystis* in a Liver Abscess: An Unusual Extraintestinal Location

**DOI:** 10.1155/crdi/7553517

**Published:** 2026-02-19

**Authors:** Daniel Rodríguez-Zúñiga, Belén Rivaya, María Vanessa López-Peláez, Bruno Antunes-Parente, Begoña Bailo, Sergio Sánchez, David Carmena

**Affiliations:** ^1^ Microbiology Department, Jove Hospital Foundation, Asturias, Gijón, Spain; ^2^ Microbiology Department, Central University Hospital of Asturias (HUCA), Asturias, Oviedo, Spain, hca.es; ^3^ Internal Medicine Department, Jove Hospital Foundation, Asturias, Gijón, Spain; ^4^ Parasitology Reference and Research Laboratory, Health Institute Carlos III, Majadahonda, Madrid, Spain, isciii.es; ^5^ Centre for Biomedical Research Network in Infectious Diseases (CIBERINFEC), Health Institute Carlos III, Madrid, Spain, isciii.es

**Keywords:** culture, dissemination, opportunistic, Spain, ST1

## Abstract

*Blastocystis* is a common anaerobic protist of uncertain pathogenicity that inhabits the human intestine. Rare cases reported in the literature suggest possible extraintestinal dissemination by unclear mechanisms. We followed a 43‐year‐old Peruvian woman presenting with low‐grade fever and gastrointestinal symptoms who was diagnosed with a liver abscess by ultrasound and CT imaging. Identification of the causative agents was achieved through a combination of mass spectrometry, molecular methods (PCR and Sanger sequencing) and culture‐based methods in blood, tissue and faecal samples. We detected gut‐associated aerobic and anaerobic bacteria, including *Escherichia coli*, *Eikenella corrodens* and *Bacteroides fragilis*. Active forms of *Blastocystis* subtype ST1 were identified in the liver abscess fluid and successfully cultured in vitro. These findings suggest translocation of gut microbiota into the portal circulation as the underlying route of infection. *Blastocystis* can act as an opportunistic invasive microorganism in locations other than the intestinal tract.

## 1. Introduction

*Blastocystis*​ is a unicellular, strictly anaerobic protist considered the most prevalent microbial eukaryote in the human intestinal tract. An estimated one billion people are colonised globally [1], suggesting that *Blastocystis* is commonly a constituent of the gut microbiota in apparently healthy individuals. Recent studies on enteric microbiota have reported associations between *Blastocystis* colonisation and greater bacterial diversity, as well as favourable cardiometabolic profiles [2]. Conversely, *Blastocystis* has also been implicated in a range of gastrointestinal symptoms, particularly abdominal pain and diarrhoea [3].

Although the large intestine constitutes the natural environment of *Blastocystis*, sporadic cases of extraintestinal dissemination have been reported. *Blastocystis*, in coinfection with *Entamoeba histolytica* or *Giardia duodenalis*, has been detected by microscopy examination in liver abscesses from two immunocompetent individuals in Taiwan [4, 5]. *Blastocystis* have also been microscopically detected in peritoneal fluid from a patient in the United States with invasive, poorly differentiated adenocarcinoma and associated bowel perforation and a patient in China with rectal signet ring cell carcinoma [6, 7]. Furthermore, *Blastocystis* subtype ST3 has been described as a potentially invasive pathogen associated with splenic cysts in a patient from Brazil [8].

In this report, we discuss the identification of *Blastocystis* in a liver abscess from a patient with altered hepatic function.

## 2. Case Presentation

A 43‐year‐old woman originating from Peru, who has been living in Spain for the past nine months, was admitted through the emergency department of our hospital after presenting with a 2‐week history of evening‐predominant fever, episodes of chills, diarrhoea, vomiting, loss of appetite and general malaise. She had a history of migraines but no other relevant medical conditions and was not on any chronic medication. She denied recent travels, and no family members reported similar symptoms. She worked as a caregiver for a person living in a household in close contact with poultry (chickens, geese) and horses, who had recently suffered from a digestive illness.

**FIGURE 1:**
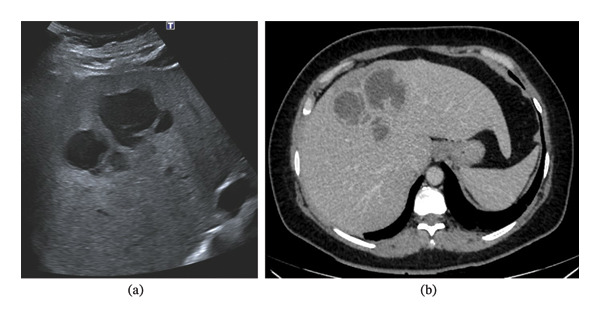
(a) On the day of admission, an abdominal ultrasound revealed three adjacent cystic formations in the hepatic dome, the largest measuring 4.8 cm with a lobulated contour, thickened wall and some septa. (b) On Day 1 after admission, an abdominal contrast‐enhanced CT showed a liver lesion affecting Segments IV and VIII measuring 9 × 8.5 × 5.5 cm.

On an initial examination, the patient had a low‐grade fever but was hemodynamically stable. Urgent lab work showed altered liver function tests (LFTs) and elevated inflammatory markers. An abdominal ultrasound was requested, revealing three adjacent cystic formations in the hepatic dome, the largest measured 4.8 cm with a lobulated contour, thickened wall and some septa (Figure 1(a)). Differential diagnoses included hydatid cysts and pyogenic or amoebic abscesses.

She was empirically treated with ceftriaxone 2 g IV every 12/24 h plus metronidazole 500 mg IV every 6 h. After 4 days of hospitalisation, she was escalated to piperacillin–tazobactam 4/0.5 g IV every 8 h as an extended infusion, due to the persistent episodes of shivering and fever. An abdominal computed tomography (CT) showed a liver lesion affecting Segments IV and VIII, measuring 9 × 8.5 × 5.5 cm with a multiloculated appearance that could correspond to a pyogenic abscess (Figure 1(b)). The possibility of a hydatid cyst could not be ruled out. Serum antibodies to *Echinococcus granulosus* were negative, but *Entamoeba histolytica* titres were positive.

Six days after admission, CT‐guided drainage yielded 270 cc of purulent fluid. The patient showed significant clinical and laboratory improvement, and a clear decrease in abscess size was observed on a follow‐up CT scan. Therefore, a shift to amoxicillin–clavulanic acid 2 g/200 mg IV every 8 h was performed.

Table 1 summarises the samples analysed and the main outcomes obtained in the different diagnostic methods used in this study. Blood cultures obtained in the emergency department yielded *Bacteroides fragilis*, and the liver abscess aspirate revealed *Escherichia coli*, *Eikenella corrodens* and *Bacteroides fragilis.* All the microorganisms were identified by MALDI‐TOF mass spectrometry, and sensitivity tests were carried out using VITEK 2 and E‐test (bioMérieux, Marcy‐l’Étoile, France). Considering the patient’s personal background and medical record, together with the CT findings, a liver abscess fluid sample was sent to the Microbiology Department of the Central University Hospital of Asturias (HUCA) for molecular parasite testing using Allplex GI‐Parasite Assay and Allplex GI‐Helminth (I) Assay (Seegene, Seoul, Korea). The test was positive for *Blastocystis* (*C*_*T*_ value: 24). The result was repeated and confirmed by a second commercial technique (BIOSYNEX Ampliquick Protozoans, Illkirch‐Graffenstaden, France), also obtaining a positive result (*C*_*T*_ value: 22). *Blastocystis*‐compatible forms were also observed in fresh smears with Lugol stain. As a result, two stool samples for molecular parasite testing were collected, providing positive results for *Blastocystis* with *C*_*T*_ values of 35 and 40, respectively.

**TABLE 1:** Samples analysed and key findings obtained from each diagnostic method used for the detection of pathogens in this study.

Sample analysed	Diagnostic method	Main outcome
Blood	MALDI‐TOF mass spectrometry	*Bacteroides fragilis*

Liver abscess aspirate	MALDI‐TOF mass spectrometry	*Escherichia coli*, *Eikenella corrodens*, *Bacteroides fragilis*
	Multiplex real‐time PCR (protozoa)^2^	*Blastocystis* (*C*_*T*_ value: 24)
	Multiplex real‐time PCR (helminths)^3^	Negative
	Multiplex real‐time PCR (protozoa)^4^	*Blastocystis* (*C*_*T*_ value: 22)
	Conventional microscopy (Lugol stain)	*Blastocystis*
	Direct PCR (genotyping)	*Blastocystis* ST1

Culture (liver abscess aspirate)	Direct PCR (genotyping)	*Blastocystis* ST1

Stool 1 (4 days posttreatment)^1^	Multiplex real‐time PCR (protozoa)^2^	*Blastocystis* (*C*_*T*_ value: 35)

Stool 2 (23 days posttreatment)^1^	Multiplex real‐time PCR (protozoa)^2^	*Blastocystis* (*C*_*T*_ value: 40)

Stool 2 (23 days posttreatment)^1^	Direct PCR (genotyping)	Negative

^1^Metronidazole 500 mg IV every 6 h.

^2^Allplex GI‐Parasite (Seegene, Seoul, Korea).

^3^Allplex GI‐Helminth (Seegene, Seoul, Korea).

^4^Ampliquick Protozoans (BIOSYNEX, Illkirch‐Graffenstaden, France).

An aliquot of the original liver abscess fluid and its corresponding eluted DNA, together with the second stool sample obtained after chemotherapeutical treatment, were shipped to the Parasitology Reference and Research Laboratory of the National Centre for Microbiology (Majadahonda, Madrid) for genotyping purposes. Extracted DNAs from all three samples were assessed in a single‐round PCR using the pan‐*Blastocystis*, barcode primer pair BhRDr/RD5 including appropriate positive (laboratory‐confirmed *Blastocystis*‐positive stool DNA) and negative (biology‐grade water) controls [9]. Additionally, the liver abscess fluid sample was successfully cultured in Jones medium following standard procedures [10]. Special care was taken during sample processing to minimise the risk of contamination in subsequent PCR and culture procedures. Sanger sequencing analyses allowed the identification of *Blastocystis* ST1 in the liver abscess fluid and its corresponding culture, but not in the stool sample. Representative sequences of these isolates have been deposited in GenBank under accession numbers PX395522 (liver abscess fluid) and PX395523 (culture).

The workup was completed with a transthoracic echocardiogram, which ruled out valvular involvement. Based on the liver abscess culture results, the patient was shifted to ceftriaxone 2 g IV every 12/24 h and metronidazole 500 mg IV every 6 h. Given her improvement, sequential oral therapy was administered, and the patient was discharged after 17 days. The patient continued treatment with cefuroxime 750 mg every 8 h and metronidazole 750 mg every 8 h for another month at home. The patient was scheduled for a routine colonoscopy and follow‐up in the outpatient clinic, which revealed colonic diverticular disease with multiple diverticula along the colon.

## 3. Discussion

*Blastocystis* is an intestinal microeukaryotic parasite that has been associated with abdominal pain and diarrhoea. Treatment is advised when symptoms are compatible and cannot be explained by the detection of another enteropathogen or a noninfectious cause. Only a few cases of *Blastocystis* identification in extraintestinal samples have been reported, including peritoneal fluid in two cancer patients [6, 7], splenic cysts [8] and liver aspirates as coinfections with *G. duodenalis* [5] or *E. histolytica* [4]. In the latter case, *E. histolytica* serology was positive and PCR confirmed coinfection, unlike our case in which serology results were more consistent with either past exposure in a patient from a highly endemic area or a possible cross‐reaction.

Liver abscesses typically have four pathophysiological mechanisms:

1. Haematogenous: By blood spread from an infectious focus, via portal (e.g., abdominal infectious processes associated or not with portal vein thrombosis, such as appendicitis or diverticulitis) or arterial (e.g., bacteraemia, endocarditis or superinfection of a haematoma, area of necrosis or ischaemia).
2. Biliar: Infections of the biliary tract (e.g., ascending cholangitis due to biliary diseases), lithiasis or previous biliary tract manipulations or surgery (e.g., biliary‐enteric diversion or biliary drainage).
3. By direct extension, after penetrating trauma or from a contiguous focus.
4. Cryptogenetic, in about 10%–15% of cases, usually related to undiagnosed sepsis.

The main risk factors described are diabetes mellitus, cirrhosis, chronic granulomatous disease, liver transplantation, alterations in the hepatobiliary‐pancreatic system and manipulations of the mucosa of the gastrointestinal tract, by surgical and nonsurgical procedures.

The detection of *Blastocystis* in a liver abscess is highly unusual, as is the isolation of *E. corrodens* at this site. By contrast, *Blastocystis*, *E. coli* and *B. fragilis* are common members of the human gut microbiota. However, the recovery of multiple intestinal aerobes and anaerobes from the liver abscess suggests an enteric source, with translocation of gut microbiota into the portal circulation as the most likely underlying route. Although the exact mechanism by which *Blastocystis* spreads beyond the intestinal tract remains unclear, two possible pathways have been proposed:

1. Opportunistic cross of the disrupted intestinal mucosal barrier as a consequence of the mechanical damage previously caused by other gut pathogens (e.g., *E. histolytica* and *G. duodenalis*) or malignancies (e.g., malignant neoplasms) [4, 5].
2. Active invasion and ulceration of the intestinal mucosa and submucosa, eventually progressing through the blood and/or lymphatic system until reaching the extraintestinal location [8].

In the present case, no evident cause (such as coinfection with an enteropathogen or a tumour) capable of disrupting the intestinal wall and facilitating the passage of *Blastocystis* into the enterohepatic circulation was identified. Instead, previous diverticular disease and a possible history of prior *E. histolytica* infection may explain the underlying pathophysiology. A previous episode of diverticulitis or *E. histolytica*‐related diarrhoea could have promoted translocation of members of the gut microbial community into the bloodstream, ultimately leading to the liver abscess. Thus, *Blastocystis* may have reached the liver in the same context as the other intestinal bacteria isolated from the abscess.

## 4. Conclusion

The potential pathogenicity of *Blastocystis*—which remains a matter of debate—would likely be linked to a polymicrobial infection involving multiple gut‐derived bacteria. The low *C*_*T*_ value obtained by RT‐PCR from the liver abscess fluid sample and its successful growth in in vitro culturing are suggestive of actively replicating *Blastocystis* cells.

## Author Contributions

Study concept and design: Daniel Rodríguez‐Zúñiga, Belén Rivaya, David Carmena. Acquisition, analysis or interpretation of data: Daniel Rodríguez‐Zúñiga, Belén Rivaya, María Vanessa López‐Peláez, Bruno Antunes‐Parente, Begoña Bailo, Sergio Sánchez. Drafting of the manuscript: Daniel Rodríguez‐Zúñiga, Belén Rivaya, Sergio Sánchez, David Carmena. Critical revision of the manuscript for important intellectual content: Daniel Rodríguez‐Zúñiga, Belén Rivaya, María Vanessa López‐Peláez, Bruno Antunes‐Parente, Begoña Bailo, Sergio Sánchez, David Carmena.

## Funding

The authors received no specific funding for this work.

## Ethics Statement

As this study was conducted within the framework of routine diagnostic practice and patient management, approval from the hospital ethics committee was not required. No identifiable personal information is disclosed in the manuscript, thereby preserving patient anonymity.

## Consent

As this study was conducted within the framework of routine diagnostic practice, individual informed consent was not required.

## Conflicts of Interest

The authors declare no conflicts of interest.

## Data Availability

All data are available within the main text.
